# Association Between Stem Anteversion and Femoral Rotation in Endoprosthetic Proximal Femoral Replacement: Insights from Two Different Prosthetic Designs

**DOI:** 10.3390/jcm14217786

**Published:** 2025-11-03

**Authors:** Tomotaka Yoshida, Hyonmin Choe, Yutaka Nezu, Yusuke Kawabata, Keiju Saito, Masanobu Takeyama, Akira Shiga, Shintaro Fujita, Naotsugu Nakajima, Naomi Kobayashi, Ken Kumagai, Hiroyuki Ike, Yutaka Inaba

**Affiliations:** 1 Department of Orthopaedic Surgery, Yokohama City University, 3-9 Fukuura, Kanazawa-ku, Yokohama City 236-0004, Kanagawa, Japan; yoshida.tom.ta@yokohama-cu.ac.jp (T.Y.);; 2 Department of Orthopaedic Surgery, Kanagawa Cancer Center, 2 Chome-3-2 Nakao, Asahi Ward, Yokohama City 241-8515, Kanagawa, Japan; 3 Department of Orthopaedic Surgery, Yokohama City University Medical Center, 4-57 Urahune-cho, Minami-ku, Yokohama City 232-0024, Kanagawa, Japan

**Keywords:** proximal femoral replacement, megaprosthesis, femoral rotation

## Abstract

**Background/Objective**: Endoprosthetic proximal femoral replacement is a reconstructive procedure for preserving ambulatory function following tumor resection. Different prosthetic systems for endoprosthetic proximal femoral replacement may result in different stem placement techniques, especially regarding the anteversion angle of the stem. The aim of this study was to evaluate femoral rotation and stem anteversion following endoprosthetic proximal femoral replacement using two different prosthetic systems, and to investigate their influence on postoperative quality of life. **Methods**: We retrospectively reviewed 30 patients who underwent endoprosthetic proximal femoral replacement at our institution between 2008 and 2022. The evaluated parameters included patient demographics, anatomical and functional stem anteversion, femoral rotation, femoral resection length, implant type, and Musculoskeletal Tumor Society score. **Results**: The cohort comprised 16 males and 14 females with a mean age of 65.2 ± 13.5 years. Twenty patients received the Global Modular Replacement System implants and 10 received the Kyocera Modular Limb Salvage System implants. The mean anatomical stem anteversion was 17.0 ± 17.7°, and the mean femoral rotation was 14.4 ± 22.6°. The Global Modular Replacement System implants demonstrated less variability in anatomical stem anteversion (11.7 ± 15.2°) compared to the Kyocera Modular Limb Salvage System (27.6 ± 18.4°, *p* = 0.02). A significant negative correlation was found between anatomical stem anteversion and femoral rotation (r = −0.78, *p* < 0.01), and a positive correlation between femoral rotation and functional stem anteversion (r = 0.62, *p* < 0.01). Musculoskeletal Tumor Society scores were available in 14 patients and correlated significantly with functional stem anteversion (r = −0.62, *p* = 0.02) and femoral resection length (r = −0.61, *p* = 0.02), but not with anatomical stem anteversion or femoral rotation alone. **Conclusions**: This study demonstrated that stem placement angles differ between prosthetic systems. These differences are attributable to variations in surgical implantation techniques and prosthesis design philosophies. In particular, the Global Modular Replacement System incorporates built-in anteversion, and when using such prostheses, referencing the linea aspera enables more stable restoration of the anatomical stem anteversion. Excessive reduction in anatomical stem anteversion is not recommended to avoid excessive external femoral rotation.

## 1. Introduction

Improved survival among patients with bone tumors and metastatic cancer has led to a rise in pathological and impending fractures of the proximal femur [1]. These fractures profoundly impair ambulatory function and quality of life (QOL), emphasizing the need for appropriate management strategies [2]. Bone metastases from cancer are frequently observed in the spine, pelvis, ribs, skull, and femur [3,4,5,6,7]. Among these, the femur is particularly at high risk for pathological fractures due to the significant mechanical stress it endures during weight-bearing and walking, including bending and torsional forces. Consequently, metastatic bone tumors in the proximal femur and the fractures associated with them are conditions that severely impair QOL [8]. Endoprosthetic proximal femoral replacement (EPFR) is a proven reconstructive technique for the management of both primary and metastatic malignant bone tumors [9,10,11]. However, there is limited literature on the functional outcomes following EPFR, particularly regarding the factors that influence limb function after surgery [12,13,14,15,16].

In EPFR for femoral bone tumors or metastasis, femoral rotation (FR) and stem anteversion (SA) are likely key factors in preventing hip dislocation and may also influence functional outcomes. However, no studies have examined how FR impacts functional stem positioning in EPFR. While several studies recommend an optimal anatomical SA of 15–20 degrees for EPFR [14,17], implant-specific positioning guidelines vary depending on the implant model. For example, the Global Modular Replacement System (GMRS) (Stryker Orthopaedics, Mahwah, NJ, USA) is designed to achieve an anatomical SA of 15 degrees when positioned perpendicular to a marker along the anterior femoral surface using the linea aspera as a reference. In contrast, the Kyocera Modular Limb Salvage System (KMLS) (Kyocera, Kyoto, Japan) lacks explicit reference markers and is typically positioned using the patella as an anterior guide to achieve an SA within 15–20 degrees. Notably, increase in FR can result in elevated functional SA, heightening the risk of posterior stem impingement [18]. However, no studies have evaluated the relationship among FR, SA, and functional outcomes in EPFR.

The purpose of this study was to compare postoperative anatomical and functional SA and FR between two different prosthetic systems for EPFR and to explore their associations with functional outcomes as measured by the Musculoskeletal Tumor Society (MSTS) score.

## 2. Materials and Methods

### 2.1. Patient Enrollment

**Figure 1:**
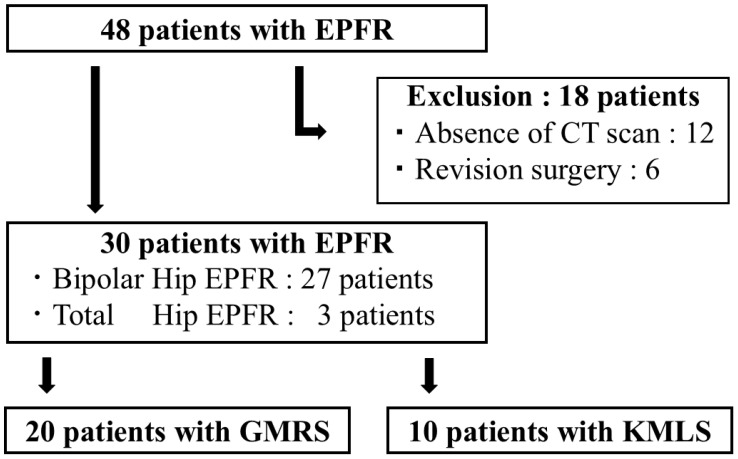
Patient enrollment in this study. The records of 48 patients who underwent Endoprosthetic proximal femoral replacement (EPFR) were reviewed. Patients who did not undergo computed tomography (CT) imaging after EPFR and those who required reoperation (18 cases) were excluded, leaving a final study cohort of 30 patients.

This retrospective study was approved by the institutional ethics review board of Yokohama City University (F220900007). The records of 48 patients who underwent EPFR in Yokohama City University Hospital from 2008 to 2022 were reviewed. Patients who did not undergo computed tomography (CT) imaging after EPFR and those who required reoperation (18 cases) were excluded, leaving a final study cohort of 30 patients (Figure 1). Comprehensive agreement for academic use of clinical information (including types of treatments, treatment progress, and other data acquired during hospitalization) was obtained from all patients at the time of their admission. No identifiable personal information is included in this manuscript. All study participants underwent a plain hip X-ray in the front view and CT scan from the first lumbar spine to the lower edge of femur. All 30 patients underwent one-stage EPFR, including 27 patients who underwent bipolar hip EPFR and 3 patients who underwent total hip EPFR (Table 1). Two types of implants were used for EPFR: the GMRS from Stryker and the KMLS from Kyocera. A total of nine orthopedic oncologists performed the surgeries included in this study. The choice of implant was primarily determined by the surgeon’s preference. In urgent cases, implant selection also depended on the availability and delivery time of the prosthesis system.

### 2.2. Surgical Techniques for EPFR in Our Institution

**Figure 2:**
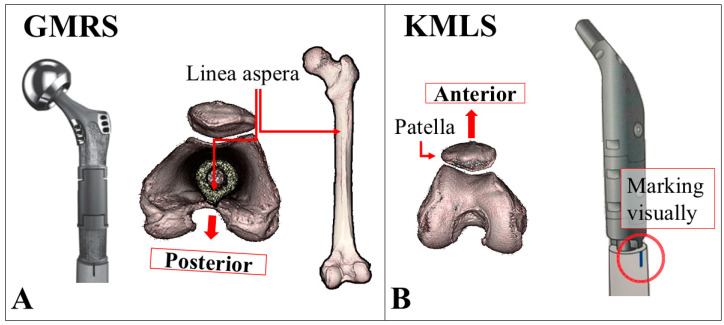
How to install each endoprosthetic proximal femoral replacement (EPFR) model. For proximal femoral arthroplasty, implant placement is described differently for each model. (**A**) Global Modular Replacement System (GMRS): This prosthesis has a built-in 15° anatomical stem anteversion at the neck. During implantation, the posterior reference is the linea aspera of the femur. The stem is positioned perpendicular to the linea aspera when the femur is viewed axially, thereby achieving a 15° anatomical anteversion. (**B**) Kyocera Modular Limb Salvage System (KMLS): This stem has no built-in anteversion. The anterior reference is the patella, and the stem is manually rotated until the neck reaches approximately 15° of anteversion relative to the patellar direction. The marking on the stem is visually aligned to confirm this orientation.

The GMRS is an anatomical stem with a built-in anatomical anteversion angle of 15 degrees at the neck. In the GMRS group, a marking was made on the anterior surface of the femur using the linea aspera as a reference. The implant was positioned perpendicular to the linea aspera to mechanically achieve an anatomical SA of 15 degrees during installation. The KMLS is a straight stem without a built-in anatomical anteversion angle at the neck. In the KMLS group, the patella was used as an anterior reference point, and the stem was positioned to achieve an anatomical SA of 15 degrees (Figure 2). All procedures were performed using the lateral approach. In both prosthesis systems, polypropylene mesh (Prolene^®^, Ethicon Inc., Somerville, NJ, USA) was wrapped around the implant, and the iliopsoas, external rotators, and abductor muscles were reattached to the mesh as much as possible to enhance joint stability and restore postoperative limb function. Dual mobility components were not used in this cohort.

### 2.3. Patient Background and Postoperative Measurements

**Figure 3:**
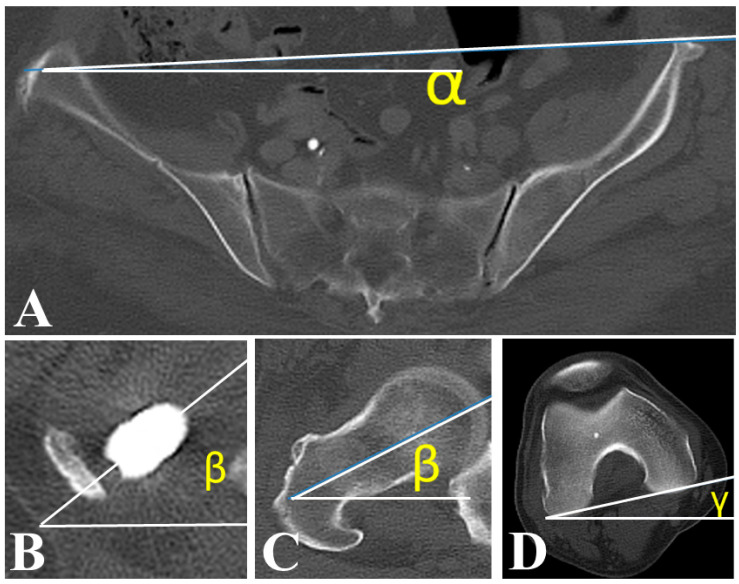
Computed tomography (CT) measurements of femoral rotation angle. (**A**) Angle α was defined as the angle formed between the CT table line and the line connecting bilateral anterior superior iliac spines. (**B**,**C**) Angle β was defined as the angle between the CT table line and the femoral neck axis. (**D**) Angle γ was defined as the angle between the CT table line and the posterior condylar line. Functional stem anteversion or external rotation angle of the femur was measured by subtracting the value for the rotation angle of the superior anterior iliac spine line from that of the stem angle (β–α) or angle of the posterior femoral condyle axis (γ–α) using a CT image acquired after surgery.

Age, sex, and type of malignancy were investigated as background characteristics. Anatomical SA, functional SA, and FR were measured using cross-sectional CT imaging after surgery (Figure 3) [18,19]. Angle α was defined as the angle between the CT table reference line and the line connecting the bilateral anterior superior iliac spines. Angle β was defined as the angle between the CT table line and the femoral neck axis. Angle γ was defined as the angle between the CT table line and the posterior condylar line. Anatomical SA was calculated as β–γ, functional SA as β–α, and FR as γ–α. All measurements were performed using dedicated PACS software (SYNAPSE Enterprise-PACS, Fujifilm, Tokyo, Japan). Postoperative QOL was assessed using the Musculoskeletal Tumor Society (MSTS) Scoring System that consists of pain, function, emotion, external support, functional independence, and gait [20]. A statistical analysis was conducted to investigate factors associated with femoral external rotation in EPFR patients.

### 2.4. Statistical Analysis

Continuous variables were compared using Student’s *t*-test when the data distribution was approximately normal. For variables with non-normal distribution, the nonparametric Mann–Whitney U test was applied. Categorical variables were analyzed using the chi-square test or Fisher’s exact test. The variance differences between the two groups were determined using Levene’s test. Correlations between continuous variables were assessed using Pearson’s correlation analysis. All analyses were performed using IBM SPSS Statistics for Windows, Version 28.0 (IBM Corp., Armonk, NY, USA). *p* < 0.05 was considered to indicate statistically significant results. All graphs were prepared using GraphPad Prism 8 software (GraphPad, Inc., La Jolla, CA, USA).

## 3. Results

The patients consisted of 16 males and 14 females, with an average age of 65.2 ± 13.5 years. The average follow-up period after EPFR was 2.1 ± 2.5 years. There were 6 cases of primary malignant bone tumors involving the hip joint (4 cases of undifferentiated pleomorphic sarcoma, 1 case of osteosarcoma, and 1 case of chondrosarcoma) and 24 cases of secondary malignant bone tumors based on the 2020 WHO classification [21]. The secondary tumors included 5 cases of renal cell carcinoma, 4 cases of prostate cancer, 4 cases of urothelial cancer (bladder, renal pelvis, or ureter), 3 cases of breast cancer, 2 cases of malignant lymphoma, 2 cases of lung cancer, and 4 cases classified as other malignancies. Twenty cases underwent EPFR with GMRS (GMRS group) and 10 cases underwent EPFR with KMLS (KMLS group).

**Figure 4:**
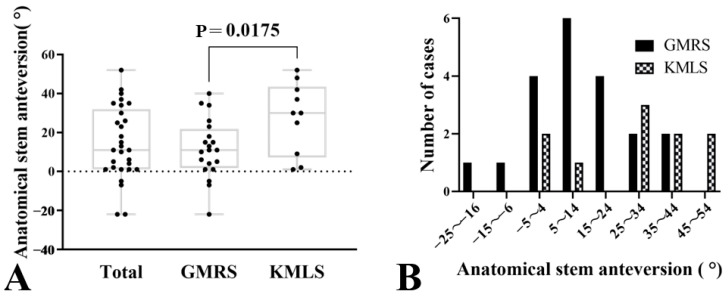
Anatomical stem anteversion (SA) on the operated side. (**A**): The mean anatomical SA of all cases was 17.0 ± 17.7° on the operated side. The range of anatomical SA on the operated side was from −22° to 52°. Additionally, the mean anatomical SA in the Global modular replacement system (GMRS) group was 11.7 ± 15.2°, compared to 27.6 ± 18.4° in the Kyocera modular limb salvage system (KMLS) group, with the KMLS group showing significantly higher anatomical SA (*p* = 0.018). (**B**): This figure presents a bar graph categorizing anatomical SA into 10° intervals. The GMRS group displayed a unimodal distribution, with most cases falling within the 5° to 14° range. In contrast, the KMLS group showed a bimodal distribution, with peaks in the −5° to 4° and 25° to 34° intervals, highlighting distinct differences in implant positioning between the two groups.

The mean anatomical femoral anteversion of all cases was 15.5 ± 7.7° on the unaffected side and the mean anatomical SA was 17.0 ± 17.7° on the operated side. The mean difference was 1.5° (95% CI: −4.9 to 7.9, *p* = 0.63), indicating no statistically significant difference. The range of anatomical femoral anteversion on the unaffected side varied from 1° to 30°, while anatomical SA on the operated side ranged from −22° to 52°. Levene’s test indicated a significant difference in variance between the two sides (F = 8.12, *p* < 0.01). Additionally, the mean anatomical SA in the GMRS group was 11.7 ± 15.2°, compared to 27.6 ± 18.4° in the KMLS group, with the KMLS group showing significantly higher anatomical SA (95% CI: −28.8 to −3.0, *p* = 0.02) (Table 2, Figure 4A). The anatomical SA in the GMRS group ranged from −22° to 40°, while it ranged from 1° to 52° in the KMLS group. The GMRS group demonstrated values closer to the target anatomical SA of 15°, although some cases exhibited retroverted anatomical SA. Figure 4B presents a bar graph categorizing anatomical SA into 10° intervals. The GMRS group displayed a unimodal distribution, with most cases falling within the 5° to 14° range. In contrast, the KMLS group showed a bimodal distribution, with peaks in the −5° to 4° and 25° to 34° intervals, highlighting distinct differences in implant positioning between the two groups (Figure 4B).

**Figure 5:**
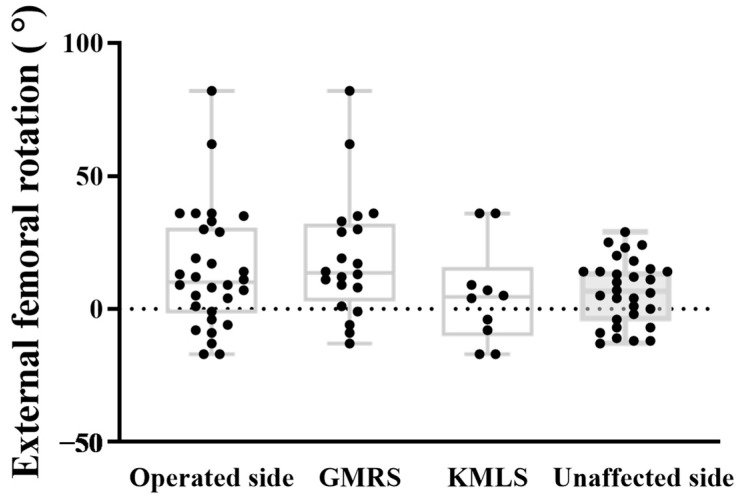
External femoral rotation (FR). The mean FR angles of all cases were 6.4°± 12.2° on the unaffected side and 14.4 ± 22.6° on the operated side, with tendency to observe a difference (*p* = 0.03). The variation on the unaffected side ranged from −13° to 29°, while the operated side ranged from −17° to 82°, showing a significant difference in variability (*p* < 0.05). The mean FR angle was 19.1 ± 23.3° in the Global modular replacement system (GMRS) group and 5.1 ± 18.7° in the Kyocera modular limb salvage system (KMLS) group, indicating greater external rotation in the GMRS group, although the difference was not statistically significant (*p* = 0.11).

**Figure 6:**
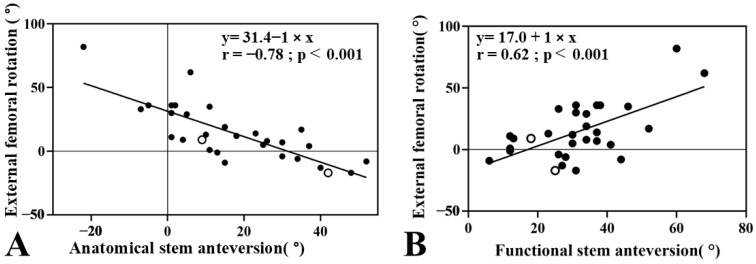
Surgical factors associated with external rotation of femur. (**A**): This figure shows relationship between anatomical stem anteversion (SA) and femoral rotation (FR) (y = 31.4 − x, r = −0.78, *p* < 0.001). A significant negative correlation was observed, indicating that greater anatomical anteversion is associated with decreased external FR, suggesting a tendency toward internal rotation of the femur. (**B**): This figure shows relationship between functional SA and FR (y = 17.0 + x, r = 0.62, *p* < 0.001). A significant positive correlation was observed, indicating that higher functional anteversion is associated with increased external FR. The blank circle indicates a case of dislocation.

The mean FR was 6.4 ± 12.2° on the unaffected side and 14.4 ± 22.6° on the operated side. A paired *t*-test revealed a significant difference in FR between sides, with the operated side showing greater external rotation (mean difference 8.0°, 95% CI: 0.9–15.2, *p* = 0.03). The variation on the unaffected side ranged from −13° to 29°, while the operated side ranged from −17° to 82°. Levene’s test revealed a significant difference in variance between the two sides (F = 5.675, *p* = 0.02). However, the mean FR was not significantly different between groups (mean difference −8.0°, 95% CI: −17.5 to 1.4, *p* = 0.094). The mean FR was 19.1 ± 23.3° in the GMRS group and 5.1 ± 18.7° in the KMLS group, indicating greater external rotation in the GMRS group. However, this difference was not statistically significant (mean difference: 14.0°, 95% CI: −3.4 to 31.4, *p* = 0.11). (Table 2, Figure 5). The analysis of femoral resection length in relation to anatomical SA, functional SA, and FR did not show a significant correlation. External FR showed a strong negative correlation with the anatomical SA (r = −0.77, 95% CI −0.89 to −0.59, *p* < 0.01) (Figure 6A) and a significant positive correlation with functional SA (r = 0.65, 95% CI 0.34 to 0.80, *p* < 0.01) (Figure 6B).

During the study period, two cases of postoperative dislocation were observed (6.7%), both of which were successfully managed with conservative treatment. Both dislocations occurred in patients who underwent total or bipolar hip arthroplasty using the KMLS implant. The Total Hip EPFR case exhibited an anatomical SA of 9 degrees and an FR angle of 9 degrees, with a functional SA of 18 degrees. Meanwhile, the bipolar hip EPFR case demonstrated an anatomical SA of 42 degrees, a FR angle of −17 degrees, and a functional SA of 25 degrees. Due to the very limited number of events, no meaningful statistical analysis could be performed. Instead, descriptive comparisons were made between dislocation and non-dislocation cases, which did not reveal clear differences in patient demographics, anatomical SA, femoral rotation angle, or functional SA.

Postoperative MSTS functional score was available for 9 cases in the GMRS group, with a mean score of 60.7 ± 20.7%, and for 5 cases in the KMLS group, with a mean score of 43.3 ± 13.1%. A Mann–Whitney U test revealed no significant difference between the two groups (U = 34.0, *p* = 0.124) (Table 2). The MSTS score was found to correlate significantly with functional SA (r = −0.62, 95% CI: −0.87 to −0.14, *p* = 0.02) and femoral resection length (r = −0.61, 95% CI: −0.86 to −0.11, *p* = 0.02), while no significant correlation was observed with patient background, anatomical SA, or FR.

## 4. Discussion

In this study, we found that the anatomical SA in EPFR patients varied greatly depending on the implant type and individual patient. The FR also varied significantly between patients. We found a significant correlation between SA and FR. Moreover, functional SA and the length of the femoral bone resection were both significantly correlated with the postoperative MSTS score. These findings suggest that accounting for FR during stem implantation—in order to achieve an optimal functional stem alignment—is crucial for maintaining good postoperative function after EPFR. Although we referred to this parameter as “functional SA” following previous reports, this value is highly posture-dependent and may vary substantially with changes in hip rotation at the time of CT acquisition. Thus, functional SA should be interpreted with caution as a surrogate marker for postoperative stem alignment.

Although an anatomical SA of 15–20° is often recommended for EPFR [14,17], no studies have directly validated this range in the context of EPFR. In our study, the anatomical SA was measured as average 17°, but the range varied widely from −22° to 52°, indicating imprecision in placement of stem in EPFR. Lackman et al. similarly demonstrated that visual assessment during stem placement in bone models resulted in anatomical SA values ranging from 1° to 50.5° [17]. This inconsistency in visually guided placement likely contributed to the variability observed in our study as well.

In our cohort, we used two implant systems (GMRS and KMLS) and compared their outcomes. Our results showed that the GMRS group achieved closer to the target SA values of 15° (mean 11.7 ± 15.2°) with less deviation compared to the KMLS group (mean 27.6 ± 18.4°). The higher deviation in the KMLS group is likely due to unstable reliance on visual assessment during positioning. Despite using the linea aspera as a stable reference in the GMRS group, inaccuracies were also noted. Previous research by Desmet et al. has reported that the position of the linea aspera varies depending on the length of bone resection [22], which may explain the cases of negative anatomical SA in our study. To ensure precise anatomical SA, preoperative imaging such as CT scans should be used to better define the position of the linea aspera as a reference point.

Internal FR is reportedly prevalent in primary total hip arthroplasty (THA), whereas external FR is more frequent in revision THA [18,19,23]. A previous study has also shown that external FR is correlated with weakened muscle strength in the psoas, iliacus, and gluteus medius muscles [18]. In our study, 19 of the 26 hips (73.1%) that underwent bipolar hip EPFR exhibited external FR. The frequent occurrence of external rotation may be attributed to the surgical disruption of muscles such as the psoas, iliacus, and gluteus medius in EPFR procedure. In our cohort, two cases experienced dislocation. Both cases involved patients with KMLS implants with anatomical SA and FR angles of 9°and 9° or 42°and −17°, respectively. Due to the very limited number of dislocation events, no meaningful statistical comparison could be made and no clear differences were observed compared with non-dislocation cases. However, external FR has previously been identified as a risk factor for dislocation in revision THA [18].

Our analysis revealed that external FR was strongly correlated with a reduction in anatomical SA (r = −0.77, *p* < 0.01) and significantly associated with an increase in functional SA (r = 0.65, *p* < 0.01). These correlations were stronger than those reported in revision THA studies [18], possibly because muscle resection in bone tumor surgery had a greater effect on the relationship between SA and FR. It should also be noted that functional SA and FR are mathematically dependent on anatomical SA. Therefore, the strong correlations observed among these parameters may partly reflect mathematical coupling rather than a purely biomechanical relationship. This limitation must be carefully considered when interpreting the clinical significance of our findings.

EPFR is a well-established treatment for both primary and metastatic malignant bone tumors [9,10,11]. However, there is limited literature on postoperative functional outcomes following EPFR for bone tumors [12,13,14,15,16]. In contrast, extensive research has been conducted on THA, emphasizing the importance of cup and SA positioning in relation to patient pelvic and lower limb posture [19,24,25]. To our knowledge, this is the first study to demonstrate the association between SA and FR in EPFR. This strong negative correlation suggests that surgeons must carefully consider anatomical SA during stem placement to avoid causing excessive FR postoperatively. According to previous reports, an anatomical SA in the range of 15° to 20° is recommended [14,17]. In the present study, a simplified relationship between anatomical SA and FR revealed a strong negative correlation (Figure 6A, FR = 31.4 − anatomical SA, r = −0.77, *p* < 0.01). This finding indicates that a decrease in anatomical SA tends to result in an externally rotated femoral orientation. Although FR itself was not directly correlated with MSTS scores in this study, excessive FR may lead to suboptimal functional SA, thereby compromising postoperative function. Therefore, maintaining the stem’s orientation close to the native anatomical alignment (i.e., avoiding overly aggressive derotation) may be important for improving postoperative outcomes after EPFR. However, this conclusion should be regarded as preliminary, and further prospective studies with larger cohorts are needed to establish a causal relationship.

This study has several limitations. First, the small sample size constitutes a significant drawback. Due to the rarity of tumor cases, prior reports focusing on SA and FR following EPFR are limited, highlighting the need for further case accumulation. Second, the evaluation was restricted to the supine position. Since FR varies between the standing and supine postures, a more comprehensive analysis incorporating standing measurements would be desirable. However, current methods to assess FR in the standing position remain limited, and establishing a three-dimensional dynamic evaluation technique is an important issue for future investigation. Third, functional SA and FR were measured only in the supine position and are strongly influenced by limb posture during CT scanning. Although foot fixation was not applied, all patients were consistently scanned in a relaxed supine position with the lower limbs placed in their natural resting alignment. This protocol was strictly maintained to reduce variability, but subtle differences in posture may still have affected measurement reproducibility. Fourth, the observed negative correlation between anatomical SA and FR may have been influenced by surgical disruption of periarticular muscles or the extent of bone resection, but these factors were not systematically evaluated. Fifth, functional SA is mathematically derived from both anatomical SA and FR; therefore, its correlation with MSTS scores may reflect confounding rather than an independent association. Sixth, surgeries were performed by different surgeons, and the potential impact of surgeon experience on stem positioning cannot be excluded. Seventh, the relatively small sample size may limit the statistical power of our analyses, and the observed associations should be interpreted with caution. Although two cases of dislocation were described, the sample size was insufficient to assess the relationship between SA/FR and dislocation. Finally, functional evaluation was restricted to the MSTS score; future studies should incorporate gait analysis and patient-reported outcomes to provide a more comprehensive assessment. Moreover, MSTS scores were available for only 14 patients. This incomplete outcome data may have introduced bias, as patients unable to complete postoperative functional assessment could differ systematically from those included. As such, the observed correlations between MSTS and alignment parameters should be interpreted with caution.

## 5. Conclusions

This study identified the relationship between SA and FR in EPFR. The group treated with the GMRS, in which the anatomical SA is mechanically predetermined, showed less variation and values closer to the target compared to the KMLS group. Our findings suggest that excessive reduction in anatomical SA should be avoided, as it may lead to excessive external FR and compromise postoperative outcomes. However, these results should be interpreted with caution due to the limited sample size and availability of functional outcome data. Further prospective, multicenter studies are required to validate these observations and to establish optimal stem orientation strategies in EPFR.

## Figures and Tables

**Table 1:** Demographic, surgical, and CT data in the EPFR cohort (n = 30).

Demographic data	Mean ± SD or n (%)
Age (years)	65.2 ± 13.5
Male, n (%)	16 (53.3)
BMI (kg/m^2^)	21.5 ± 4.7
Diagnosis (hips)	Primary: 6 (20), Secondary: 24 (80)
Surgical data	
Surgical procedure (hips)	Total hip: 3 (10), Bipolar hip: 27 (90)
Femoral resection length (cm)	14.3 ± 3.9
CT data	
Postoperative anatomical SA (°)	17.0 ± 17.7
Postoperative FR (°)	14.4 ± 22.6

Data are presented as mean ± standard deviation (SD) or number (%). BMI, body mass index; SA, stem anteversion; FR, femoral rotation.

**Table 2:** Comparison of demographic, surgical, and CT data between GMRS and KMLS groups in the EPFR cohort.

	GMRS (n = 20)	KMLS (n = 10)	Mean Difference (95% CI)	*p*-Value
Demographic data				
Age (years)	65.0 ± 13.6	63.4 ± 12.7	1.6 (−8.2 to 11.4)	0.87
Male, n (%)	12 (60)	4 (40)		0.44 ^1^
BMI (kg/m^2^)	21.8 ± 5.2	21.0 ± 4.3	0.8 (−3.4 to 5.0)	0.70
Diagnosis (hips)	1ry: 4, 2ndry: 16	1ry: 1, 2ndry: 9		0.64 ^1^
ASA ≥ III, n (%)	2 (10)	1 (10)		1.00 ^1^
Pathological fracture, n (%)	13 (65)	6 (60)		1.00 ^1^
Radiotherapy, n (%)	3 (15)	3 (30)		1.00 ^1^
Surgical data				
Surgical procedure (hips)	Total hip: 1, Bipolar hip: 19	Total hip: 2, Bipolar hip: 8		0.25 ^1^
Femoral resection length (cm)	13.8 ± 3.6	15.4 ± 4.8	−1.6 (−4.6 to 1.4)	0.28
CT data				
Postoperative anatomical SA (°)	11.7 ± 15.2	27.6 ± 18.4	−15.6 (−28.8 to −3.0)	0.02
Postoperative FR (°)	19.1 ± 23.3	5.1 ± 18.7	14.0 (−3.4 to 31.4)	0.11
Complications				
Infection, n (%)	5 (25)	2 (20)		1.00 ^1^
Dislocation, n (%)	0 (0)	2 (20)		0.10 ^1^

Data are presented as mean ± standard deviation (SD) or number (%). BMI, body mass index; SA, stem anteversion; FR, femoral rotation. ^1^ Fisher’s exact test was used when expected counts <5; otherwise, Chi-square test.

## Data Availability

The data presented in this study are available on reasonable request from the corresponding author. The data are not publicly available due to privacy and ethical restrictions.
